# Men's perception of current and ideal body composition and the influence of media internalization on body judgements

**DOI:** 10.3389/fpsyg.2023.1116686

**Published:** 2023-05-02

**Authors:** Vicki Groves, Bethany J. Ridley, Piers L. Cornelissen, Nadia Maalin, Sophie Mohamed, Robin S. S. Kramer, Kristofor McCarty, Martin J. Tovée, Katri K. Cornelissen

**Affiliations:** ^1^Department of Psychology, Northumbria University, Newcastle upon Tyne, United Kingdom; ^2^Department of Psychology, Birmingham City University, Birmingham, United Kingdom; ^3^Aberdeen Royal Infirmary, NHS Grampian, Aberdeen, United Kingdom; ^4^School of Psychology, University of Lincoln, Lincoln, United Kingdom

**Keywords:** male body image, muscle, fat, media influences, media internalization

## Abstract

**Introduction:**

To determine men's body ideals and the factors that influence these choices, this study used a matrix of computer generated (CG) male bodies (based on an analysis of 3D scanned real bodies) which independently varied in fat and muscle content.

**Methods:**

Two hundred and fifty-eight male participants completed a range of psychometric measures to index body concerns and body ideal internalization and then chose the CG body that best reflected their own current body, as well as the body that reflected their personal ideal. A subset of participants was then retested to check that these judgements were stable over time.

**Results:**

While judgements of the ideal body seem to be influenced by a shared appearance ideal, the degree to which this ideal was internalized showed significant variability between participants. The effect of this internalization was reflected in the difference between the estimated current body and the ideal.

**Discussion:**

Higher internalization led to a preference for higher muscle and lower fat content. This preference was most marked for fat content, although reducing adiposity also made the underlying musculature more salient. Additionally, the ideal body composition was modulated by the composition the participant believed his current body had (i.e., it seemed that a participant's ideal body was anchored by what they believed to be their current body and what change was possible from this starting point).

## 1. Introduction

The term “body image” is applied to the internal representation we have of our bodies. This includes not just its physical dimensions, but also a person's feelings, perceptions, thoughts, and beliefs about their body (Cash and Deagle, 1997). Body image is an important concern for men as well as women (e.g., Edwards et al., 2016; Quittkat et al., 2019). Poor male body image, aspiring to an unrealistic body size and shape while at the same time denigrating the size and shape of your actual body, has previously been associated with the development of a range of negative psychological outcomes including eating disordered behaviors and depression (Kanayama et al., 2006; Tylka, 2011).

A key determinant of body image is thought to be the emphasis placed on ideal body shape within a given cultural context. The tripartite influence model provides a powerful sociocultural account of how such cultural ideals are spread and popularized within a population. The model seeks to explain the social pressure someone can experience from family, peers, and the media (Thompson et al., 1999; Shroff and Thompson, 2006). Each person will be exposed to different amounts of these pressures and will also vary in the degree to which they feel these pressures to apply to themselves. It is the impact of these pressures which is suggested to mediate between the body ideal propagated by society and the induction of body image dissatisfaction in an individual (Keery et al., 2004; Schaefer et al., 2015). Therefore, body dissatisfaction arises because an individual feels that they are significantly different from this ideal, but still feel that they should achieve it (i.e., they see a marked difference between the body they have and the body they think they should have).

In Western society, there is a strong focus on an idealized physical appearance which is promulgated through both the media and social media, creating a strong pressure to conform to a body ideal that for most men is unachievable. This male ideal is defined by a well-developed upper body to create a V-shaped torso and low body fat, so the underlying musculature is clearly visible (Leit et al., 2002; Ridgeway and Tylka, 2005; McCreary et al., 2007; Murray et al., 2017; Mohamed et al., 2021). This idealized body shape has been spread by male models in magazines (Frederick et al., 2005; Lanzieri and Cook, 2013), in film (Pope et al., 2000), computer game characters (Martins et al., 2011), and action figures (Baghurst et al., 2006). This ideal is reinforced by the fitspiration images of highly muscled, low-fat bodies on social media (Carrotte et al., 2017; Tiggemann and Zaccardo, 2018). Exposure to this media content increases the probability that men will undertake excessive exercise and abuse anabolic steroids, which may have negative health consequences (Cafri et al., 2006; Horwitz et al., 2019; Mossman and Pacey, 2019; Tiggemann and Anderberg, 2020). This drive for muscularity can lead to the development of anorexia nervosa (AN) (Klimek et al., 2018) or the onset of muscle dysmorphia (sometimes referred to as reverse AN) (Pope et al., 2000). It is perhaps, unsurprising that the incidence of AN in men seems to be rising (Strother et al., 2012; Sweeting et al., 2015). Under such circumstances, the study of body ideals in male populations and the factors that shape these ideals is therefore important. Additionally, it is important to have body stimuli that independently vary muscle and fat. The drive for body changes in men is less about increasing or decreasing overall body weight but increasing muscularity and decreasing fat content. Stimulus sets that only vary in one dimension (usually trying to simulate Body Mass Index (BMI) change), will not capture this percept as both muscle and fat will be covaried (see below). Thus, for this study we have developed a stimulus in which muscle and fat vary independently.

### 1.1. Why use stimuli varying in body composition?

Previous studies testing male body image judgements have usually used stimuli varying either in BMI as a proxy for fat content, or body shape (such as the shoulder-to-waist ratio; SWR) as a measure of muscularity (e.g., Maisey et al., 1999; Fan et al., 2005; Hönekopp et al., 2007). However, both sets of measures are potentially unreliable. BMI is not an accurate index of fat content (see Gardner and Brown, 2010; Ridley et al., 2022). It is based on both fat tissue and skeletal muscle (Sturman et al., 2017). If the muscle and fat levels of a large sample of men are plotted against one another, they will be positively correlated. But if this data is separated out into their BMI categories, then within each BMI, the correlation becomes negative (Maalin et al., 2021; Ridley et al., 2022). This is called Simpson's paradox (Simpson, 1951). The result of this effect is that a number of body compositions can have the same BMI but not have the same shape (Yajnik and Yudkin, 2004; Mullie et al., 2008).

This is also true of other measures of body shape such as SWR. The same body shape can have significantly different ratios of fat and muscle (see Ridley et al., 2022). Therefore, it cannot be assumed that a specific shape is indicative of a specific composition as has been the case in past studies (e.g., Maisey et al., 1999; Fan et al., 2005; Hönekopp et al., 2007; Sell et al., 2017). Consequently, in this study we have used 3D body shape scan data together with body composition measurements to guide the manipulation of body composition in our stimuli. We created Computer Generated Imagery (CGI) images using the technique detailed by Maalin et al. (2021). The resultant stimulus set illustrates 2D body composition space. One of the dimensions indexes fat variation, and the other dimension, orthogonal to the first, indexes variation in muscle mass.

### 1.2. The current study

**Figure 1:**
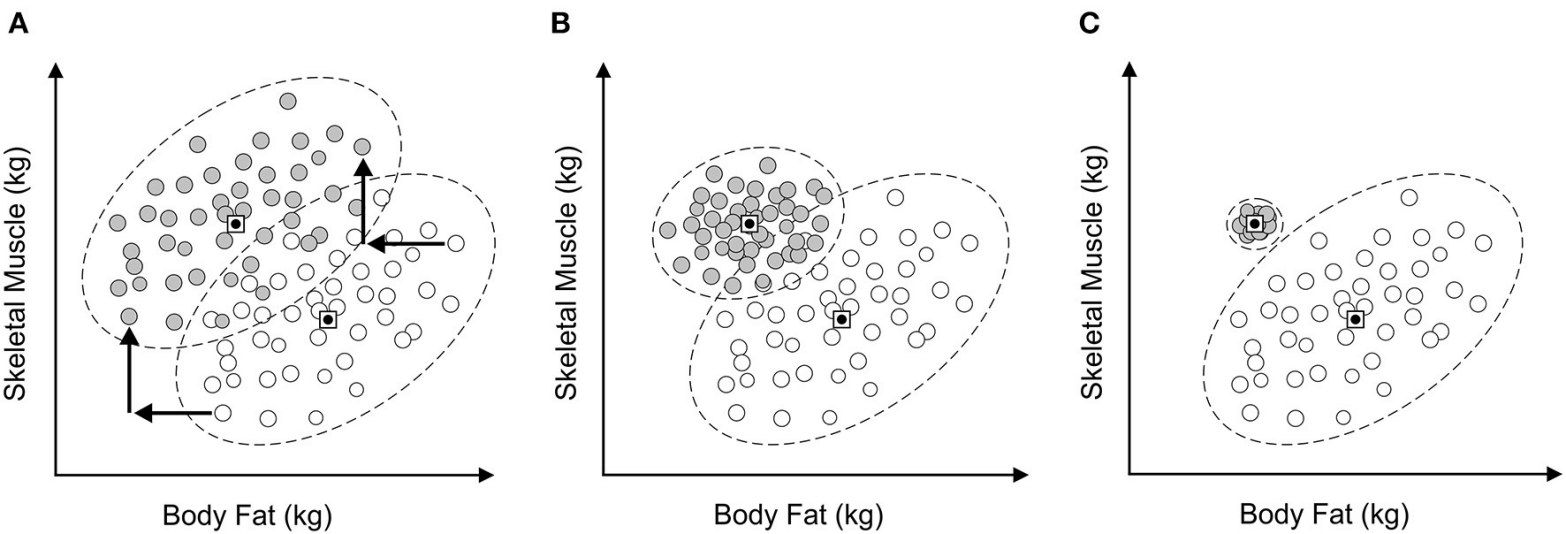
**(A–C)** Three alternative ways that estimates of current body composition and body ideals are potentially related in 2D muscle-fat space. White circles represent estimates of body composition, filled circles represent ideal body composition.

Although it is possible to show that a given society promulgates a consistent muscular ideal, for men, what is actually internalized by individuals within the population may vary substantially. So, the impact of this cultural ideal is not necessarily uniform. Suppose one asks a large sample of men to illustrate the body shape and size they believe they have, as well as the body shape and size they want. Figure 1 shows, in principle, three ways that current beliefs about body shape and associated ideals could be related to each other. In each scatterplot, an individual's current and ideal body are plotted in body composition space. Across Figures 1A–C, the average body composition of the current beliefs and ideals are the same (i.e., the squares with dots) in each case. However, in Figure 1A, each current belief has been translated to an ideal belief by a fixed increase in muscle mass and a fixed decrease in fat. It is as if the concept of an ideal has been operationalized as a desire for a fixed change in body composition irrespective of the starting point. The opposite extreme is represented in Figure 1C, where all ideals converge on a point, as if an identical ideal from the environment has been internalized by all individuals, all of whom want to achieve the same singular result. Any residual variation in composition around this point would be attributable to measurement error only. Figure 1B represents an intermediate position. Here, there is a tendency to converge toward a single point, but individual differences, perhaps in attitudes to muscularity and fat content as well as measurement error, lead to variation in the distribution of ideal body shapes in body composition space. *In the current study, we ask which of these three alternatives best fits the data*. We accept that these alternatives are to a certain extent straw men, but this allows us to frame our argument within a clear theoretical framework with explicit outcomes.

To do this, we used a matrix of CGI male bodies based on an analysis of 3D scanned real bodies, which independently varied in fat and muscle content. Using the matrix, participants were asked to choose “the body that best reflected their own current body” and then “the body that best reflected their ideal.” They were also asked to complete a range of psychometric measures to index body concerns and appearance internalization. We then retested a subset of participants to check that the judgement was stable over time. We addressed three questions to test the validity of our approach.

#### 1.2.1. Three tests of the validity of our approach

First, we asked whether the average body composition for men's current beliefs about their body shape in our sample was consistent with the median body composition for men in the UK. To do this, we compared our sample's body composition to data from the UK Biobank (Lee et al., 2020). This showed good agreement, suggesting that our sample was reasonably representative of men in the UK.

Second, a number of previous studies have suggested that body size estimation is predicted by two independent components: (i) a visual bias in the way size is judged (contraction bias) and (ii) the observer's psychological profile including how an observer feels about their shape and size, their self-esteem and mood (Cornelissen et al., 2015, 2016, 2017; Irvine et al., 2019).

Contraction bias is caused by the way object size is judged. When estimating the size of an object of a particular class, an observer uses an internal perceptual template, based on the average of all the previous examples of that object class, as a yard stick against which to make their judgement (Poulton, 1989). As a result, judgements are most precise when an estimation is of an object is close in size to the internal template. As the disparity in size between the template and the estimated object increases, then so does inaccuracy of the judgement. The object is thought to nearer in size to the template than it actually is (i.e. larger objects are underestimated and smaller objects are overestimated). This visual bias also applies to body size judgements, including judgements of one's own body. The effect of contraction bias can be graphically illustrated by plotting the estimation of personal body size against the actual body size, then the resultant plotted function will have a slope of <1. This is because someone with a BMI below the population average will think they are larger than they are, whereas someone with a BMI above this average will think they are smaller than they are (Poulton, 1989; Cornelissen et al., 2016).

Additionally, the second factor that impacts on the accuracy of estimates is the observer's psychological concerns. For a given observer BMI, increasing psychological concerns will also raise the degree of personal size over-estimation. In our previous studies we have indexed these concerns using a number of psychometric questionnaires including the Body Shape Questionnaire (BSQ-16b; Evans and Dolan, 1993), Eating Disorders Examination Questionnaire (EDEQ; Fairburn and Beglin, 1994), Beck Depression Inventory (BDI; Beck et al., 1961), and Rosenberg Self-Esteem Scale (RSE; Rosenberg, 1965). Therefore, we tested whether the use of our somatomorphic matrix task replicates this finding.

Third, as this study was conducted online, we do not have actual body composition measurements for each participant. However, previous studies using similar techniques in online studies have validated their measures by correlating their participants' psychometric scores against the fat and muscle discrepancy scores obtained from performance in the somatomorphic matrix (e.g., Talbot et al., 2019a,b). Here, we do the same.

#### 1.2.2. The main research questions

We carried out a multivariate multiple regression analysis of current vs. ideal body composition judgements to address two questions: (1) Which of the three models of the relationship between current and ideal body composition, as represented in Figure 1, is most appropriate? (2) In the event that either Figure 1A or Figure 1B is a good description of this relationship, we also ask: to what extent do participants' perceptions of their own current bodies and the degree to which they internalize media ideals predict individual differences in the location of their chosen ideals in 2D fat-muscle (body composition) space?

## 2. Methods

### 2.1. Ethics

Ethical approval for this study was given by the Department of Psychology ethical committee at Northumbria University.

### 2.2. Sample size

In a recent study, Ridley et al. (2022) assumed that asking participants to identify the body composition of male bodies that they found most attractive was a good proxy measure for the ideal body. In Study 2 of their paper, Ridley et al. (2022) found that internalization of athletic/muscular ideals, as indexed by the Sociocultural Attitudes Toward Appearance Questionnaire-4 (SATAQ-4), predicted the muscle mass of these selected ideals with an effect size, *f*^2^ = 0.045. Using this effect size, at an alpha = 0.05 and a power = 0.9, G^*^Power returns a required sample size for a multiple regression model with up to 5 predictors of *n* = 235 (Faul et al., 2009).

### 2.3. Participants

We recruited 302 men aged 18–64 through the Prolific survey platform using opportunity sampling. Before the study began, potential participants were informed that they were not eligible to take part if they had an eating disorder. Of the 302 who did take part, 44 participants were excluded due to a combination of poor calibration (see below) and/or failure to complete the whole study, resulting in a final sample of 258 participants with complete datasets. This left a total of 258 participants with complete datasets. Participants were advised that, due to the nature of the study, only males as assigned at birth could take part; there were no further exclusion criteria. Participants were remunerated by the Prolific fair standard hourly pay for 30 min of participation.

To ensure that our participants were engaging with the online task and providing accurate body judgement choices, participants also performed a calibration check using data from a 3 x 3 matrix of calibration squares, presented prior to the matrix task. Participants simply had to click on the middle of each of the 9 squares as accurately as they could. Twenty participants' responses fell outside the ±1% error in either the horizontal of vertical direction for at least one or more calibration squares. They were therefore removed from further analysis.

### 2.4. Psychometric measures

#### 2.4.1. Male Body Attitudes Scale

The Male Body Attitudes Scale (MBAS) measures three subscales: muscularity satisfaction, low body fat attitudes and perceptions, and height satisfaction (Tylka et al., 2005). As our focus for this study was on body composition, we retained only the body fat and muscularity subscales. For this study, Cronbach's alpha was 0.91.

#### 2.4.2. Eating Disorders Examination Questionnaire

The EDEQ contains 28 items and four subscales; the Restraint subscale considers control of eating, the Eating concern subscale measures preoccupation with food and social eating, the Shape concerns subscale indexes body shape dissatisfaction, and the Weight concerns subscale indexes weight dissatisfaction (Fairburn and Beglin, 2008). A global score for the questionnaire is produced by averaging the appropriate items, with a higher score indicating higher disordered eating pathology. For this study, Cronbach's alpha for the EDEQ was 0.93.

#### 2.4.3. Sociocultural Attitudes Toward Appearance Questionnaire-4

The SATAQ-4 is a 22-item measure that measures the internalization of appearance ideals and related pressures (Schaefer et al., 2015). There are five sub-scales: two for the internalization of ideals (thin/low body fat and athletic/muscular) and three for pressures (media, peers, and family). Individual questions are scored on a five-point Likert scale with response options ranging from 1 (definitely disagree) to 5 (definitely agree). Higher scores suggest higher internalization and acceptance of society's appearance ideals, and rising pressures from family, peers, and media. For this study, Cronbach's alpha was 0.88.

#### 2.4.4. Multidimensional Perfectionism Scale (MPS)

This 45-item questionnaire has three subscales: self-oriented, other oriented, and socially prescribed perfectionism; using a Likert scale ranging from 1 (disagree) to 7 (agree) (Hewitt and Flett, 1990). Subscale values are calculated separately with high scores for each indicating higher negative perfectionistic attitudes and behaviors. Scoring information for this questionnaire indicates that other-oriented perfectionism relates to the way you perceive others or how they may perceive you, rather than self-assessment. Therefore, in the current study, we retained only self and socially oriented perfectionism scores as these constructs have been described as having a strong link to eating disordered behavior (Bardone-Cone et al., 2007) and negative body image in men (Grammas and Schwartz, 2009). For this study, Cronbach's alpha was 0.87.

#### 2.4.5. Beck Depression Inventory

The 21-item BDI questionnaire, has scores ranging from 0 to 3 on each item, with higher scores indicating greater levels of depressive feelings (Beck et al., 1961). The total score ranges from 0 to 63, with scores <10, 11–16, 17–20, 21–30, 31–40, and >40 representative of normal, mild mood disturbance, borderline clinical depression, moderate depression, severe depression, and extreme depression, respectively. For this study, Cronbach's alpha was 0.91.

### 2.5. Anthropometric measurements

As this study was conducted online, we could not take an accurate measure of participant body composition. We therefore asked participants to provide their approximate height in centimeters (cm), and their approximate weight in kilograms (kg). This allowed us to calculate participant BMI.

### 2.6. The body matrix

**Figure 2:**
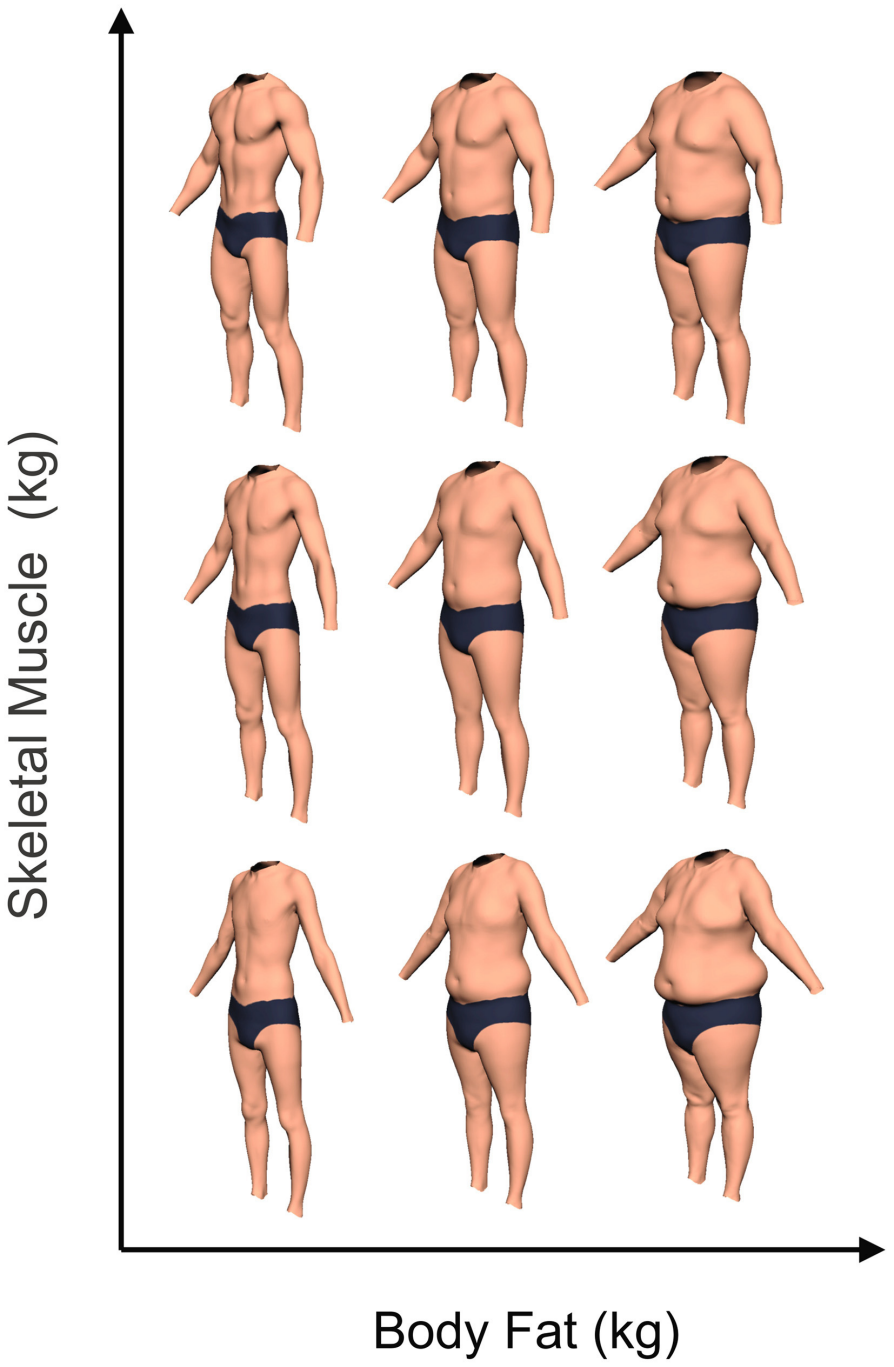
Examples of the CGI body stimuli used in this study illustrating how the bodies change as body composition varies.

In a previous study, we collected high-resolution 3D body shape scans and bioimpedance measures from 176 men (Maalin et al., 2021). We then calculated how body composition predicts body shape. Using this relationship, we can produce anthropometrically accurate CGI bodies whose size and shape reflect the changes caused by changing body composition. The body matrix is composed of 32 of these CGI bodies independently varying in muscle and fat. Muscle content was varied between 0 and 55 kg (in 7.5 kg steps) and fat content was varied between 0 and 44 kg (in 5.5 kg steps). The bodies were presented in three-quarter view (see Figure 2).

The matrix was presented online using PsychoPy and the PsychoJS (javascript) library (Peirce et al., 2019), hosted on pavlovia.org. To address the issue of variable screen size and resolution across different participants, the matrix was scaled using height relative units on PsychoPy which converts pixel resolution, i.e., the height of the screen would be measured from 1 = top, 0 = center, and −1 = bottom of the screen. Using this scaling technique, the height of the matrix was set at 98% of the height of the screen, and the width adjusted appropriately (maintaining aspect ratio). To test that the presentation of the matrix worked consistently in this way, prior to the body composition measurements, participants completed a calibration squares task in which a series of nine squares were clicked on with the mouse cursor to precisely log the edges and the center of the image. Since the software was able to recognize the screen resolution, we could test how accurately participants clicked on the squares. As outlined in the participants section, data were rejected if calibration errors exceeded ±1% of the screen height or screen width.

Participants were asked to use a desktop or laptop computer using Chrome or Firefox, and to run the task in full screen. All the bodies appeared on screen at the same time in a matrix, and the participant selected a body from the array. Participants were asked to select the position in the 2D body space represented by the body matrix that best represents (i) your current body size and shape, and (ii) your ideal body size and shape. When completing the current/ideal task, the coordinates of where they left clicked the pointer device were logged using the height relative units. Actual and ideal judgements were made five times each, and presentation of the current/ideal task were alternated, with the final body composition measurement being calculated as an average of all five judgements.

### 2.7. Procedure

Participants provided informed consent before proceeding with the study. Age, height, and weight of participants were collected. Participants then completed the psychometric questionnaires in full before being directed to Pavlovia.org to complete the body image matrix task. Following completion, participants returned to the Qualtrics survey for debriefing. The procedure lasted around 30 min.

#### 2.7.1. Analysis pipeline

- Descriptive statistics for study sample.
- Comparison of the mean and 95% CI values for skeletal muscle mass and body fat for men's current beliefs about body shape compared to UK body composition norms.
- Calculation of the difference in body fat and skeletal muscle mass between men's ideal body shape and the body shape they currently believe they have. Correlations of these differences with psychometric measures of satisfaction with muscularity, eating disorder and depressive symptomatology, sociocultural attitudes, and perfectionism.
- Multiple regression of self-estimated BMI on actual BMI. To implement this, it required two preliminary steps. The first step required a principal component analysis of psychometric scores to reveal a single PSYCH component. This is a combination of the attitudes which contribute to body size disturbance: disturbed attitudes to eating, weight, and shape, depressive symptomatology, tendency to internalize family, peer, and media pressures for thinness and muscularity. The second step required conversion of current beliefs about body composition to self-estimates of BMI, using a calibration equation derived from Maalin et al. (2021).
- Multivariate multiple regression model predicting participants' ideal body composition from the body composition they believe they currently have. This model controls for a number of covariates including participants' age, and their scores on the BDI, EDEQ, MBAS fat, MBAS muscle, SATAQ, and perfectionism psychometric tasks.
- Seven to fourteen-day test-retest reliability of the Matrix task.

## 3. Results

### 3.1. Univariate statistics

Of the 258 adult male participants who provided complete datasets that were adequately calibrated, 2.55% identified as Asian, 6.55% Black, 4.0% Hispanic, 0.73% Other, and 86.18% White. Table 1 shows the descriptive statistics for all participant characteristics and psychometric scores, as well as the average fat and muscle values for the estimated current bodies and the ideal bodies. Also included is the difference between the current and ideal totafat values (fat discrepancy) and skeletal muscle mass values (muscle discrepancy).

**Table 1:** Range, means and standard deviations showing participant characteristics and psychometric scores for the final sample (*N* = 258).

**Characteristics**	***M (SD)***	**Range**
Age (years)	25.01 (7.38)	18–64
BMI (weight/height^2^)	24.39 (4.39)	14.88–39.18
EDEQ global score	1.54 (1.16)	0–5.06
MBAS fat	40.30 (13.60)	13–71
MBAS muscle	47.21 (13.25)	17–83
SATAQ fat internalization	13.50 (4.29)	5–25
SATAQ muscle internalization	15.00 (4.53)	5–25
SATAQ family pressure	8.25 (4.29)	4–20
SATAQ peer pressure	7.53 (3.85)	4–18
SATAQ media pressure	10.17 (5.19)	4–20
BDI	11.06 (8.80)	0–46
Self-perfectionism	62.54 (12.4)	15–89
Social perfectionism	62.45 (10.42)	15–88
Current body fat choice (kg)	17.36 (11.26)	0.79–51.71
Ideal body fat choice (kg)	11.73 (7.82)	0.41–49.96
Body fat discrepancy (kg)	5.63 (9.29)	−24.44 to 33.82
Current muscle choice (kg)	36.14 (11.09)	7–68.08
Ideal muscle choice (kg)	49.82 (8.86)	24.79–70.14
Muscle discrepancy (kg)	13.68 (12.69)	−16.42 to 57.67

### 3.2. Comparison between current beliefs and UK body composition norms

The UK Biobank reports body composition measures for 375,512 White participants (Maalin et al., 2021). The muscle and fat composition of the body on the 50th Centile of this data set has values for total skeletal muscle mass of 37 kg and body fat mass of 19 kg. Table 2 shows that the 95% confidence interval for our sample's current beliefs about their total body fat mass is within 250 g of the UK Biobank 50th centile for men (Maalin et al., 2021). The 95% confidence interval for their current beliefs about their skeletal muscle mass include the UK Biobank 50th centile. Based on this result, we suggest that our sample is reasonably representative of men in the UK.

**Table 2:** Body composition for current and ideal beliefs in our male sample (*N* = 258), compared to the UK Biobank 50th centile for men's body composition.

	**Total body fat (kg)**			**Skeletal muscle mass (kg)**		
	**Median**	**M**	**95% CI**	**Median**	**M**	**95% CI**
UK biobank	19.0			37.0		
Current belief		17.36	15.98–18.74		36.14	34.78–37.50
Ideal		11.73	10.77–12.69		49.82	48.73–50.91

### 3.3. Correlations between fat and muscle discrepancy and psychometric task performance

Talbot et al. (2019b) reported correlations between MBAS and EDEQ scores and the fat and muscle discrepancies they measured using their somatomorphic matrix task in 2,733 men. They reported significant positive correlations between MBAS body fat scores and body fat discrepancy *(r* = 0.66), as well as MBAS muscularity and muscle discrepancy (*r* = 0.44). They also reported a significant, positive correlation between EDEQ and fat discrepancy (*r* = 0.48). For our study, we found significant positive correlations between MBAS body fat scores and fat discrepancy (*r* = 0.54, *p* < 0.0001), as well as MBAS muscularity and muscle discrepancy (*r* = 0.32, *p* < 0.0001). However, we also found a small but significant correlation between MBAS muscularity and fat discrepancy (*r* = 0.17, *p* = 0.006). Like Talbot et al. (2019b), we found a positive correlation between EDEQ and fat discrepancy (*r* = 0.40, *p* < 0.0001). No other correlations between these variables were statistically significant.

### 3.4. Self-estimated BMI as a function of actual BMI

In the multivariate analysis, we wanted to determine whether the results of previous studies (Cornelissen et al., 2015, 2016, 2017; Irvine et al., 2019) could be replicated, using the current stimulus set. Specifically, whether a regression of self-estimated BMI on actual BMI showed: (a) a perceptual contraction bias, and (b) an independent contribution to self-estimated BMI from participants' psychometric performance. To prevent adding variance inflation, we initially tested for co-linearity amongst the psychometric variables.

Given that Table 3 shows substantial and significant Pearson correlations between EDEQ, MBAS fat, the SATAQ sub-scores, and other attitudinal measures including BDI and Perfectionism, we therefore used PROC FACTOR in SASv9.4 (SAS Institute, North Carolina, US) to carry out a principal components analysis with varimax rotation to identify the significant latent variable(s) in the psychometric data. The factor scores from these latent variable(s) were used in the statistical models. The Kaiser–Meyer–Olkin (KMO) measure of sampling adequacy (which indicates the degree of diffusion in the pattern of correlations) was 0.80, suggesting an acceptable sample. One factor had an eigenvalue greater than Kaiser's criterion of 1 (i.e., 3.83), which explained 80% of the variance. The scree plot showed an inflection, i.e., Cattel's criterion which also justified retaining just the one factor. The residuals were all small, and the overall root mean square off-diagonal residual was 0.08, indicating that the factor structure explained most of the correlations. The factor loadings for EDEQ, MBAS fat, MBAS muscle, Fat internalization, Peer pressure, Family pressure, Media pressure, BDI, Social perfectionism, Self-perfectionism, and Muscle internalization, were: 0.85, 0.79, 0.63, 0.62, 0.62, 0.58, 0.54, 0.50, 0.42, 0.31, and 0.29. This latent variable, which we call PSYCH, is a combination of the attitudes which contribute to body size disturbance: disturbed attitudes to eating, weight, and shape, depressive symptomatology, tendency to internalize family, peer, and media pressures for thinness and muscularity.

**Table 3:** Pearson correlations between demographic and psychometric variables.

	**Age**	**BMI**	**BDI**	**EDEQ**	**MBAS**	**MBAS**	**Fat**	**Muscle**	**Family**	**Peer**	**Media**	**Self**
					**Fat**	**Muscle**	**Int**.	**Int**.	**Pressure**	**Pressure**	**Pressure**	**Perf**.
BMI	0.23^**^											
BDI	−0.096	0.065										
EDEQ	−0.036	0.39^***^	0.47^***^									
MBAS fat	0.021	0.56^***^	0.43^***^	0.79^***^								
MBAS muscle	−0.14^*^	−0.022	0.46^***^	0.50^***^	0.59^***^							
Fat internalization	−0.13^*^	0.18^*^	0.23^**^	0.53^***^	0.54^***^	0.35^***^						
Muscle internalization	−0.14^*^	−0.10	0.040	0.23^**^	0.12	0.26^***^	0.31^***^					
Family pressure	−0.0029	0.32^***^	0.28^***^	0.50^***^	0.48^***^	0.28^***^	0.31^***^	0.058				
Peer pressure	−0.16^*^	0.19^*^	0.28^***^	0.52^***^	0.43^***^	0.35^***^	0.38^***^	0.16^*^	0.56^***^			
Media pressure	−0.099	0.14^*^	0.30^***^	0.43^***^	0.35^***^	0.26^***^	0.40^***^	0.17^*^	0.35^***^	0.35^***^		
Self perfectionism	−0.077	−0.12	0.02	0.18^*^	0.12	0.15^*^	0.27^***^	0.25^***^	0.14^*^	0.14^*^	0.26^***^	
Social perfectionism	−0.075	0.078	0.22^**^	0.24^***^	0.19^*^	0.33^***^	0.22^**^	0.20^**^	0.22^**^	0.24^***^	0.34^***^	0.58^***^

^*^p < 0.05; ^**^p < 0.001; ^***^p < 0.0001.

Next, we used the body composition data from the 176 adult males in Maalin et al. (2021) to construct an equation that allowed us to convert body composition into self-estimates of BMI. The equation was:


$$\begin{array}{l} y=\beta_{0}+\beta_{1}.x_{1}+\beta_{2}.x_{2}+\;\beta_{3}.x_{3} \end{array}$$

where *y* = self-estimated BMI, β_0_ = intercept (12.088), β_1_ = regression weight for fat tissue mass (i.e., 0.403), β_2_ = regression weight for skeletal muscle mass (i.e., 0.170), β_3_ = regression weight for age (i.e., 0.023).

**Figure 3:**
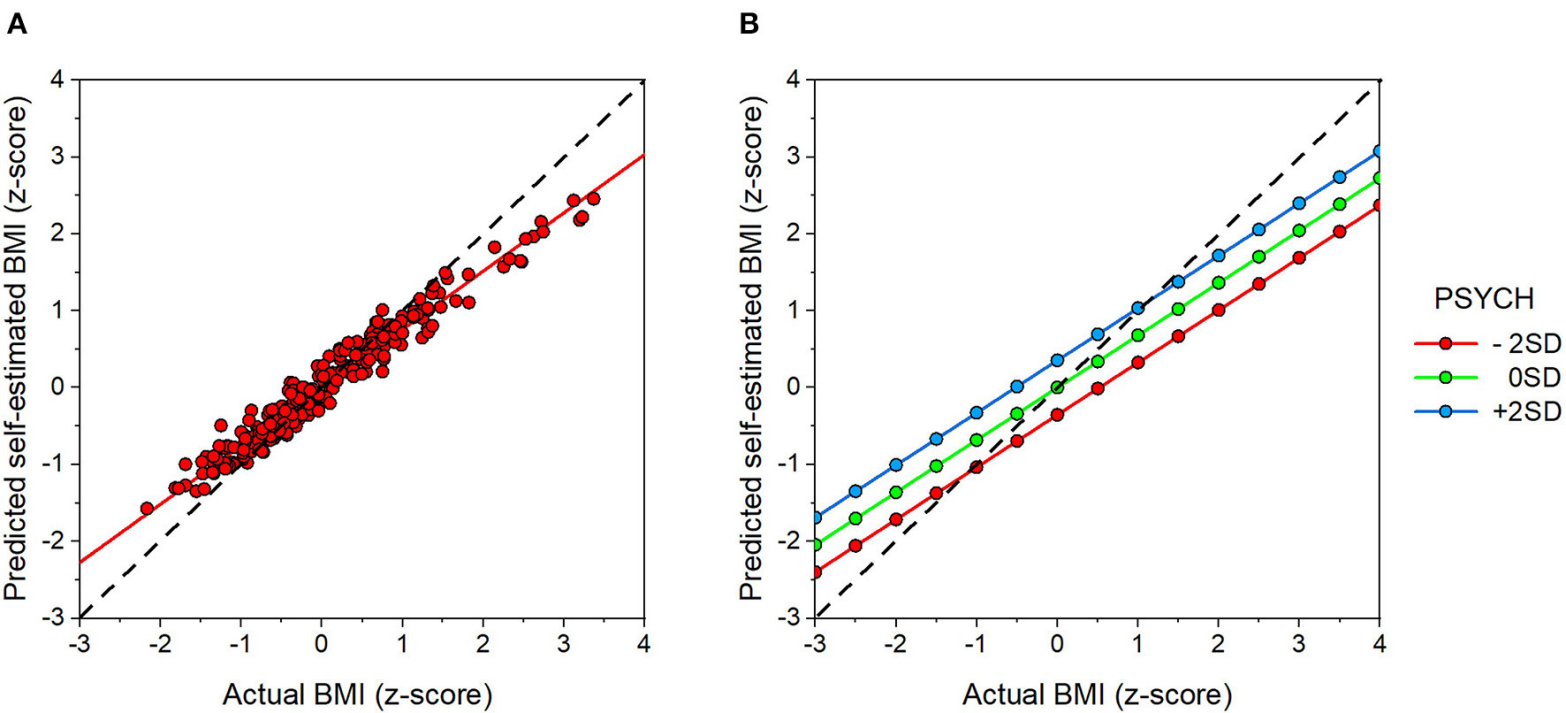
**(A)** Shows a scatter plot of predicted self-estimated BMI, derived from the body composition that participants chose in the matrix task, plotted as a function of their self-reported actual BMI. Both sets of variables were centered prior to modeling. **(B)** Shows the independent effect of PSYCH (red = −2sd, green = 0 sd, and blue = + 2sd) on the regression of self-estimated BMI as a function actual BMI. At any actual BMI, increased psychological distress about one's body, indexed by increasing PSYCH scores, leads to an increase in self-estimated BMI.

Next PROC REG in SAS (v9.4) was used to run a multiple regression model to predict self-estimated BMI from actual BMI, PSYCH, and age. The model explained 60.1% of the variance in self-estimated BMI and showed statistically significant positive effects of BMI (*t* = 15.58, *p* < 0.0001, β = 0.68, *SE* = 0.043), PSYCH (*t* = 3.88, *p* = 0.0001, β = 0.18, *SE* = 0.045), and age (*t* = 2.25, *p* = 0.03, β = 0.093, *SE* = 0.041). There were no statistically significant interaction terms in the model. Critically, the regression weight for actual BMI was significantly <1 (*F*1, 254 = 53.13, *p* < 0.0001). Figure 3 is a graphical illustration of the model outcome. Figure 3A clearly shows the positive relationship between BMI and self-estimated BMI with a slope <1, consistent with contraction bias. Figure 3B illustrates the independent contribution from PSYCH whereby individuals with greater psychological concerns about their body give responses which lead to higher self-estimated BMI.

### 3.5. Ideal body shape

In the second multivariate analysis, we modeled the relationship between the body shape that participants would ideally like to have and the body shape they believe they currently have. Table 3 shows the means for total fat and skeletal muscle mass, together with their respective 95% confidence intervals, separately for the “current” and “ideal” body judgements. We used PROC GLM (SAS v9.4) to compute a multivariate test of condition (i.e., “ideal” vs. “current”) on body composition. Pillai's Trace test for the overall effect of condition was statistically significant, *V* = 0.37, *F*_(2, 513)_ = 153.14, *p* < 0.0001.

The matrix task we used resulted in a multivariate dataset with two outcome measures (i.e., male image fat, and male image muscularity) together with a number of explanatory variables including: the rater's “current” body composition and their age, as well as their psychometric performance (BDI, EDEQ, MBAS fat, MBAS muscle, Fat internalization, Muscle internalization, Family pressure, Peer pressure, Media pressure, Self-perfectionism and Social perfectionism). Owing to the repeated measures multivariate design, PROC MIXED in SAS v9.4 (SAS Institute, North Carolina, USA) was used to compute multivariate multiple regression models. To do this, we generated a class variable (named Var in this paper) to identify the two levels of the outcome measure. The Var variable generates two design matrix columns corresponding to two intercept terms, one for each outcome. Therefore, we also used the NOINT option in the MODEL statement to prevent PROC MIXED from generating another, unnecessary intercept column. In general, Var is crossed with each other effect in the model. The REPEATED statement specified an unstructured covariance matrix between the two responses. For model optimization, we started with an empty model, and added explanatory variables provided they significantly reduced the −2 Log Likelihood. Table 4 shows the results of the fixed effects for the full multivariate multiple regression model, selected on this basis. We found significant covariance for participant intercepts (*Z* = 11.36, *p* < 0.0001) as well as between the two responses (*Z* = 11.36, *p* < 0.0001). The final model explained 87.9% of the variance in ideal body composition judgements relative to the unexplained variance in ideal body composition judgements.

**Table 4:** Outcome from the final multivariate multiple regression model.

**Fixed effects**	**Var**	**Estimate**	**SE**	***t*-value**	***p*-value**	**−2 LL**
Empty model						4588.7
Full model						3475.6
Var	Ideal body fat	7.82	1.92	4.06	<0.0001	
	Ideal muscle	36.82	2.49	14.81	<0.0001	
Var × current body fat	Ideal body fat	0.52	0.049	10.66	<0.0001	
	Ideal Muscle	−0.24	0.063	−3.85	0.0002	
Var × current muscle	Ideal body fat	−0.012	0.038	−0.33	0.7	
	Ideal Muscle	0.17	0.049	3.51	0.0005	
Var × muscle int	Ideal body fat	0.042	0.092	0.45	0.7	
	Ideal muscle	0.47	0.12	3.91	0.0001	
Var × MBAS fat	Ideal body fat	−0.17	0.043	−3.88	0.0001	
	Ideal muscle	0.12	0.055	2.15	0.03	
Var × BDI	Ideal body fat	0.12	0.050	2.45	0.02	
	Ideal muscle	−0.069	0.065	−1.06	0.3	

We also calculated several additional multivariate tests. Pillai's Trace showed a statistically significant main effect of “current” estimated fat mass, *V* = 0.32, *F*_(2, 251)_ = 59.76, *p* < 0.0001, and “current” muscle mass, *V* = 0.046, *F*_(2, 251)_ = 6.00, *p* = 0.003, on “ideal” body composition. In addition, there were statistically significant main effects of MBAS fat, *V* = 0.067, *F*_(2, 251)_ = 8.99, *p* = 0.0002, Muscle internalization, *V* = 0.058, *F*_(2, 251)_ = 7.74, *p* = 0.0005, and BDI, *V* = 0.025, *F*_(2, 251)_ = 3.28, *p* = 0.04.

**Figure 4:**
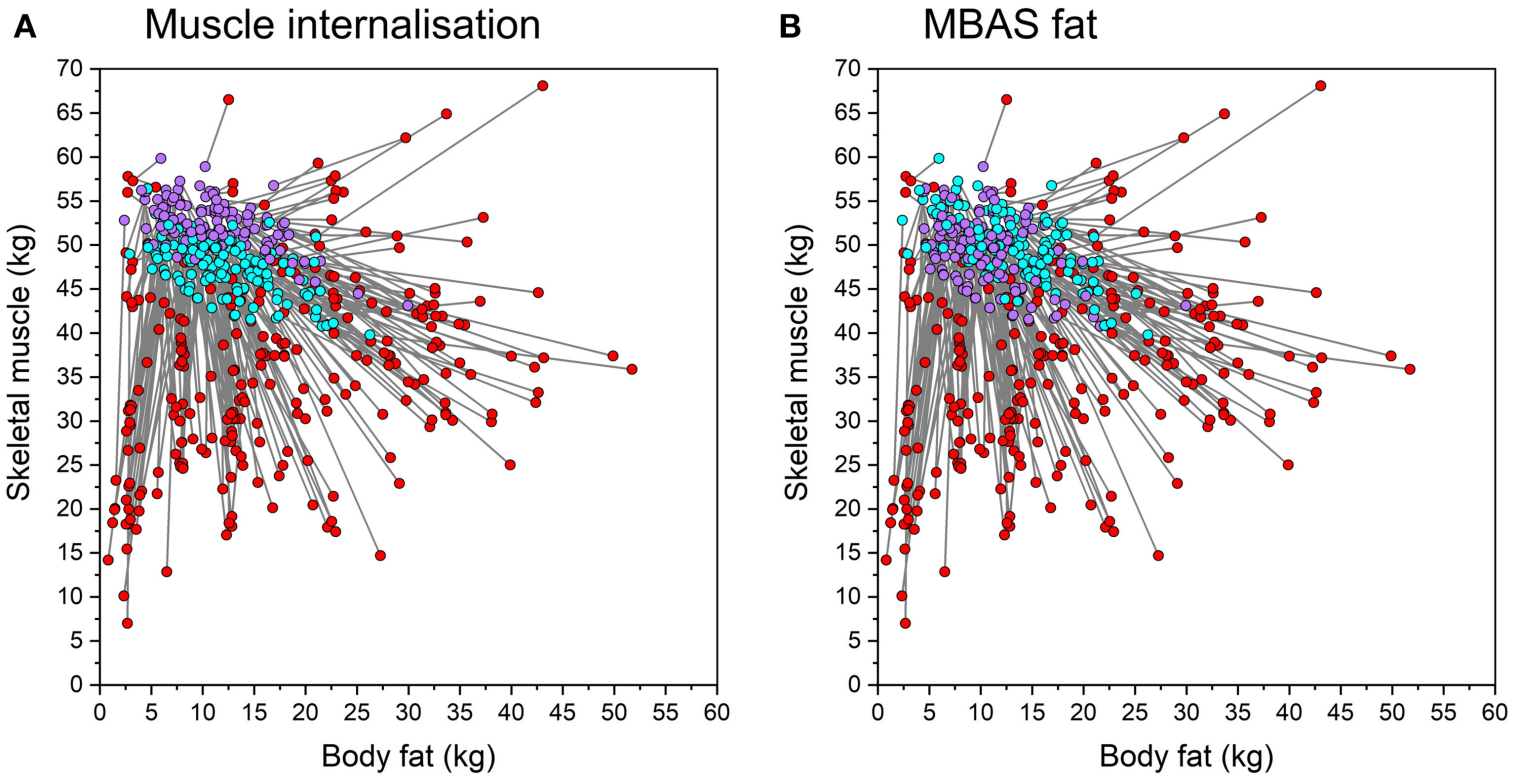
Vector plots of “current” estimate of body composition (red circles) and ideal body composition predicted from the multivariate multiple regression model in Table 4. The lines connect each individual participant's “current” belief to their ideal. The ideal points are split into two distributions based on their psychometric score. **(A)** Ideal body composition data points from individuals with a SATAQ muscle internalization score below the sample median are color coded in cyan, above the median in violet. **(B)** Ideal body composition data points from individuals with a MBAS fat score below the sample median are color coded in cyan, above the median in violet.

The relationship between “current” body composition and “ideal” body composition, which is captured by the statistical model in Table 4, is illustrated graphically by the vector plots in Figure 4. In addition, the influence of muscle internalization and MBAS fat on the ideal body composition choices are also illustrated in Figures 4A, B. Overall, Figure 4 shows a contraction of the widely dispersed envelope around “current” body composition beliefs (muscle mass *M* = 36.14, *SD* = 11.09; fat mass *M* = 17.36, *SD* = 11.26) toward a much more constrained ideal body composition envelope, which has higher muscle mass and lower fat, on average (muscle mass *M* = 49.82, *SD* = 8.86; fat mass *M* = 11.73, *SD* = 7.82). This contraction from the “current” space to the “ideal” space is emphasized by the vector plot which connects individual participants' beliefs about their “current” body composition to their “ideal.” Moreover, the color coding of the data points in the “ideal” space reveals the influence of muscle internalization in Figure 4A and MBAS fat in Figure 4B. In the former case, the more that participants have internalized information about muscularity, the higher their desired muscle mass. In the latter case, higher concerns about fat are expressed as lower total adiposity choices in the “ideal.”

**Figure 5:**
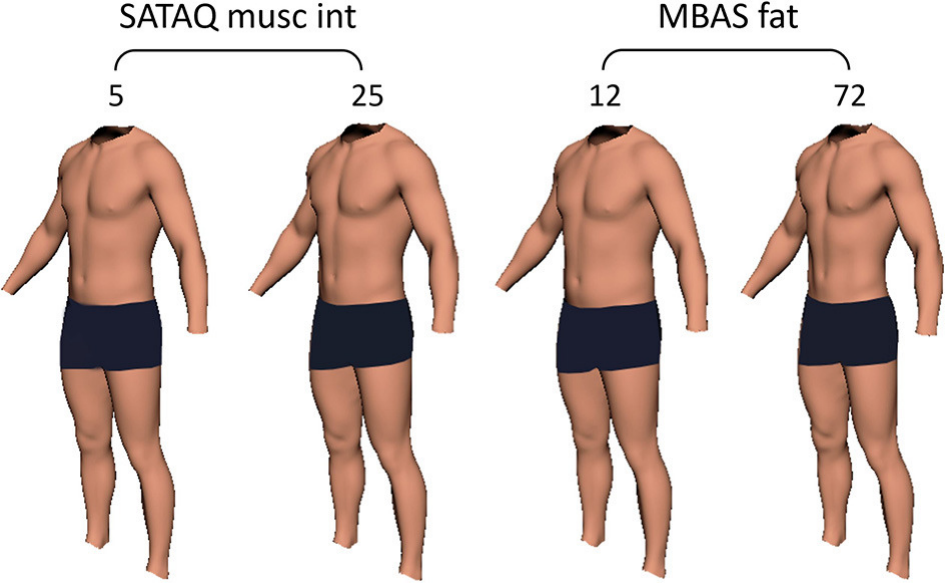
Illustrations of male bodies corresponding to the SATAQ muscle internalization scores of 5 (minimum) and 25 (maximum), as well as the MBAS fat scores of 12 (minimum) and 72 (maximum). In each case all other parameters from the model in Table 4 were held at their respective sample means. Using the modeling technique of Maalin et al. (2021), set to the nearest 1 kg increment, when the observer has a SATAQ score of 5 and 25, total fat mass is 11 and 12 kg, respectively, and skeletal muscle mass is 45 and 54 kg, respectively. When the observer has an MBAS fat score of 12 and 72, total fat mass is 16 and 6 kg, respectively, and skeletal muscle mass is 46 and 53 kg, respectively.

To further visualize the outcome of the model in Table 4, we computed the LSMEAN total fat mass and skeletal muscle mass values for the “ideal” at the minimum (5) and maximum (25) SATAQ muscle internalization scores, as well as the minimum (12) and maximum (72) for MBAS fat scores, respectively. The bodies representing these four states are shown in Figure 5. On inspection, the fat mass change attributable to the full range of MBAS fat scale is visually salient. By comparison, the skeletal muscle mass change attributed to the full range of SATAQ muscle internalization, while statistically significant, is less salient.

### 3.6. Test-retest reliability

As a final step, 31 men (aged 18–63, *M* = 28.97, *SD* = 10.99) were recruited into a reduced version of the study to explore the test-retest reliability of the matrix task over a 7–14-day period. On two occasions, separated by at least 7 days but no longer than 14 days, participants were asked to: select the area on the presented body matrix that best represents (i) your current body size and shape (five selections), and (ii) your ideal body size and shape (five selections). Current and ideal judgements were randomized, with the final body composition measurement for both current and ideal being computed as an average of all five judgements. Participants were recruited through the Prolific participant pool. For timepoint 1, participants were first directed to the Qualtrics platform where they provided informed consent before proceeding with the study. Their age, height, weight, and ethnicity were collected. Participants were then directed to Pavlovia.org to complete the new body matrix. Seven days after timepoint 1, participants were contacted with the link to the new body matrix for timepoint 2 and were asked to complete this within seven days. During this session, participants were taken directly to Pavlovia.org to complete the body size judgement task. Each session took ~10 min to complete. Table 5 shows the mean scores for the test-retest measurements of body composition, as well as the correlation between the two time points, which suggest good reliability.

**Table 5:** Mean (SD) scores and Pearson correlation coefficients for “current,” “ideal” body composition judgements at each timepoint.

		**Timepoint 1, M (SD)**	**Timepoint 2, M (SD)**	**Pearson *r***
Body fat tissue (kg)	Current	22.84 (12.13)	21.36 (10.10)	0.78^***^
	Ideal	15.56 (12.11)	14.67 (9.73)	0.65^**^
Skeletal muscle (kg)	Current	35.56 (9.38)	37.44 (11.06)	0.74^***^
	Ideal	47.11 (10.81)	48.92 (8.54)	0.69^***^

^**^p < 0.001, ^***^p < 0.0001.

## 4. Discussion

### 4.1. Estimates of current body size

As this study was online, it was not possible to physically measure fat and muscle values for each participant. However, previous studies using similar techniques in online studies have validated their measures by correlating their participants' psychometric scores against the fat and muscle discrepancy scores derived from the visual test (e.g., Talbot et al., 2019a,b). Our results showed strong correlations in the expected directions for the participants' psychometric scores with the fat and muscle discrepancy scores using matrix style stimuli, and these results are in keeping with those reported by Talbot et al. (2019a,b).

Participants reported their height and weight, allowing us to compare their actual BMI with the BMI of the body they have chosen as their current body from the test matrix. The pattern of results in this comparison is consistent with previous studies (Cornelissen et al., 2016, 2017) and shows the same two independent component model: (a) a perceptual component, captured by a visual bias (contraction bias), and (b) an attitudinal component, captured by the PSYCH variable. Contraction bias results from an observer using an internal visual template for an object class (such as bodies) as yard stick against which to judge the size of other bodies (Poulton, 1989). The judgement is most accurate when the body being judged is close in size to the template body, but the accuracy decreases as the size disparity between template and the judged body becomes larger. So larger bodies will be seen as smaller than their actual size, and smaller bodies will be seen as larger. As a result, an example smaller in size than the reference will be overestimated and an example larger will be underestimated (Winkler and Rhodes, 2005; Cornelissen et al., 2013). Consistent with the phenomenon of contraction bias, the slope of the regression of self-estimated BMI on actual BMI is <1, with a rotation point, with respect to the line of equality, at around the mean actual BMI for men in the UK (Health Survey for England, 2012). Previous studies in which participants have estimated their body size using other methods such as rating sets of bodies or through methods of adjustment tasks have shown the same contraction bias (e.g., Cornelissen et al., 2015, 2022). This suggests that the way our participants are using the body matrix is producing the same pattern of results as other techniques. Additionally, the attitudinal component (represented by the PSYCH variable in this study) also modulates the estimates independently, with individuals with greater psychological concerns about their fat content reporting higher body adiposity in the task (see Figure 3B), as previous studies have also found (Cornelissen et al., 2016, 2017).

In this study, participants' average estimated current body composition was also very similar to that reported for the average UK male population. For example, the 50th centile for male skeletal muscle mass is 37 kg and fat mass is 19 kg, based on 375,512 White men from the UK Biobank (Lee et al., 2020). This is very similar to the average estimated body composition of our participants reported here using the matrix stimulus, and both the Biobank muscle and fat levels fall within the 95% confidence limits for the average current body reported here (see Table 2), suggesting that the range of body compositions chosen by the participants in this study using the matrix is representative of the range of male body composition in the UK (Lee et al., 2020). By contrast, the average ideal body composition is significantly different from the estimated current composition and the Biobank values fall outside the 95% confidence limits for the average ideal composition (see Table 2).

### 4.2. Internalization of the body ideal

As expected, there was a wide distribution in the current body estimates in 2D muscle-fat space, reflecting the diversity in a normal male population (Figure 4). Although most of the participants wanted to increase muscle and decrease fat content in their ideal relative to their estimated body composition, this was not always the case (see Figure 4). Instead, the participants' ideal choices seem to converge on a restricted range of muscle and fat values consistent with a generally common high muscle, low fat ideal. However, this does not seem to be such a tight distribution converging around a single ideal point for all our participants, as would be consistent with a single shared cultural ideal as the driver for body preferences (as illustrated in Figure 1C). We can also reject the simplest hypothesis which is a fixed reduction in fat and fixed increase in muscle, where the amount of muscle increases and fat decreases by the same value across all the men (Figure 1A). Instead, it seems a looser distribution, more consistent with the hypothesis illustrated in Figure 1B, in which multiple factors including both current body composition and appearance internalization play a role. Interestingly, the degree of internalization of the muscle and fat ideals modulate how extreme the ideal body composition will be (Figure 5). Higher internalization leads to a preference for a significantly higher muscle and lower fat content. However, the degree of modulation differs between muscle and fat. As illustrated in Figure 5, the degree of muscle variation between the highest and lowest muscle internalization is quite subtle. By contrast, the variation in fat content with the degree of adiposity internalization is far more obvious.

It has been suggested that muscle dissatisfaction and fat dissatisfaction represent separate pathways to develop body size and shape, with dissociable effects on engagement in muscle behavior and disordered eating (Tylka, 2011). Men who are dissatisfied with their muscularity show a greater probability of body building (Cafri et al., 2005; Goldfield et al., 2006; Thompson and Cafri, 2007), and men who are dissatisfied with their fat levels show a greater probability of disordered eating to reduce their fat (Tylka et al., 2005).

Previous studies have suggested that about 90% of men would like to be more muscular and ~40% would like to have less fat (Frederick et al., 2006, 2007). This is consistent with our results, where although there is a widespread preference amongst our participants for a body with high muscularity, there is more variation in preferences for fat between participants. As mentioned above, Figure 5 illustrates that there is little visible change in body muscularity as SATAQ muscle internalization varies, but the change in fat and subsequent muscle definition appear more visibly significant with variation in MBAS fat. It is possible that fat plays a role in men's body judgements because it potentially masks muscle tone (Hildebrandt et al., 2004; Ridgeway and Tylka, 2005; Tylka et al., 2005; Frederick et al., 2007). Men do not seem to want to lose weight or be thin, as increased muscularity usually leads to increased body size and weight (Ridgeway and Tylka, 2005). Instead, they want the improved muscle definition that goes with lower fat content. Thus, the preference for lower fat might be seen as arising as a secondary effect of a desire to be appear more muscular, although a reduction in fat and increased muscle may be operationalized through different behaviors (e.g., exercise versus dietary behaviors).

### 4.3. Independent variation of muscle and fat content

This study used a matrix of body stimuli which were directly calibrated using the relationship between 3D body shape and composition. Previous studies using a similar approach have also tried to construct matrices showing a range of muscle and fat combinations (e.g., Talbot et al., 2019a,b; Arkenau et al., 2020). However, questions remain regarding the calibration of these somatomorphic matrices. One approach has been to use CGI to create photorealistic stimulus sets (e.g., Cho and Lee, 2013; Arkenau et al., 2020). This is a choice partially driven by the poor quality of the line-drawn versions (e.g., Gruber et al., 2000; Hildebrandt et al., 2004). These CGI modeling packages seem to allow the muscle and fat content of a CG body to be independently varied, but these changes are not calibrated to anthropometric measurements and instead are an artist's impression of how these dimensions change which is a serious limitation for these stimulus sets. To try to counter this limitation, Talbot et al. (2019a) modeled their CGI stimuli after an existing line-drawn somatomorphic matrix (Gruber et al., 2000). The bodies in this original matrix were designed to illustrate specific fat-free mass indices (FFMIs) and body fat percentages (BF%) (Gruber et al., 2000). To create these figures, the FFMI and BF% values of a group of volunteers were measured using skinfold calipers and then the volunteers were photographed. Next these photographs were used as a reference for a set of line-drawings depicting the relationship between body shape and composition (Gruber et al., 2000). The original matrix was composed of 100 line-drawn silhouettes (Gruber et al., 2000), which was then cut down to 34 drawings (Cafri and Thompson, 2004), although this reduced version has low reliability (Cafri et al., 2004). These drawings have been criticized for being unsatisfactory depictions of body composition and show little detail about the underlying muscularity (Talbot et al., 2019b). Talbot et al. (2019a) produced a new matrix composed of CGI bodies using the line drawings as a template (Talbot et al., 2019a,b). This new matrix added simulated muscle tone and shape, but these additions had no anthropometric basis. There is no clear mapping of actual body composition onto the size, shape, and appearance of the stimuli in the matrices. As a result, studies using this matrix represent an innovative approach to the issue of body judgements but whose results can only be regarded as provisional.

In our study, the use of stimuli which were directly generated from a 3D scan database (Maalin et al., 2021) means that we could put precise values on the fat and muscle composition of the current and ideal body estimations and determine the physical and psychometric factors which predicted these judgements. Nevertheless, the current approach is not without its own limitations. First, the size of the sample of scanned bodies used by Maalin et al. (2021) would ideally be larger and cover a wider range of body composition. Second, the statistical modeling does not treat skeletal structure, body fat, and skeletal muscle mass entirely independently. Consequently, there is a degree of covariation particularly between skeletal structure and muscle mass.

Finally, an additional advantage of the current study was the fact that we were able to calibrate the responses that participants made on the particular device they were using to carry out the study. In so doing, we could exclude responses derived from inaccurate cursor selection, and be sure that participants were clicking on the mouse location in the matrix task that they intended.

### 4.4. Limitations and future directions

This was an online study which allowed the collection of a large sample of male participants, but this prevented the collection of direct anthropometric measures. Although participants self-reported their weight and height, this may not be as accurate as direct, in-person measures (e.g., Wang et al., 2002; Gorber et al., 2007). Most importantly, although we have made an estimate of each participant's body composition, ideally this would have been measured directly. Future studies should consider this approach to address the role of personal composition in influencing body judgements. Practical considerations also limited in the amount of biographical information we could take from each participant. A more detailed characterization of each participant's mental health history, socioeconomic status and psychological profile may provide additional insight into influences on body judgements. Additionally, we excluded men with an eating disorder in this study, but ultimately, we need to know the preferences of these groups to accurately measure their pathology. Future studies should focus on specific male eating disordered populations to accurately characterize their preferences with stimuli that independently vary muscle sand fat.

## 5. Conclusion

Our results suggest that, while judgements of the ideal body are influenced by a shared appearance ideal, the degree to which this ideal is internalized shows variability. This is reflected in the extent to which the current body is changed to create the ideal. Higher internalization leads to a preference for higher muscle and lower fat content. This is most marked for fat content, although reducing fat content also makes the underling muscles more visible. However, this does not seem to be an entirely abstract ideal, as the preferred composition outcome is modulated by the composition that the participant believes his current body has (i.e., what is possible from his current starting point under perfect circumstances).

## Data availability statement

The raw data supporting the conclusions of this article will be made available by the authors, without undue reservation.

## Ethics statement

The studies involving human participants were reviewed and approved by Department of Psychology Ethical Committee at Northumbria University. The patients/participants provided their written informed consent to participate in this study.

## Author contributions

VG, KC, and PC designed the study. NM, SM, BR, MT, and RK created the stimuli. KM programmed the studies. VG collected data. PC, VG, and RK analyzed the data. MT, VG, and PC wrote the first draft of the manuscript. All authors contributed to manuscript revision, read, and approved the submitted version.
